# Role of nurses in moderating the emotional dynamics in the clinical learning environments: Implications for medical students' experience

**DOI:** 10.1111/medu.15728

**Published:** 2025-06-02

**Authors:** Shalini Gupta, Stella Howden, Mandy Moffat, Lindsey Pope, Cate Kennedy

**Affiliations:** ^1^ School of Medicine University of Dundee Dundee Scotland; ^2^ Learning and Teaching Academy Herriot‐Watt University Edinburgh UK; ^3^ College of Medicine University of Glasgow Medical School Glasgow Scotland

## Abstract

**Introduction:**

Existing literature recognises that health professionals' socialisation in the workplace involves an emotional component, including management of feelings as per professional expectations and demands. However, there is limited understanding of the emotions‐related processes involved in the interprofessional educational space of the clinical learning environment (CLE) and the role of nurses in moderating the emotional dynamics. This paper explores ways in which emotions operate in the interprofessional encounters of the CLE, utilising Hochschild's theory on emotional work.

**Methods:**

An ethnographic approach was adopted that included 120 hours of observations conducted in two hospital wards hosting clinical placements for medical students and 36 individual interviews with staff and students populating these clinical sites. Key themes were derived using the circular process of ethnographic data analysis utilising the sensitising concepts of emotional labour, emotional gifts and feeling rules, together with the relational dimensions of interprofessional interactions in the CLE.

**Results:**

The following key themes were identified: emotional gifts from nurses to medical students and junior doctors, interprofessional interactions suggesting a breach in feeling rules and suspension of interprofessional hierarchies that engendered goodwill and appreciation among health care teams in the CLE. Interconnected subthemes highlight the nurses' efforts to maintain the sentimental order in the CLE through generously gifting emotional support to students and doctors in a variety of situations, impacting their professional socialisation and well‐being. However, workplace stresses such as time pressures and staff shortages created interprofessional tensions, generating negative emotions. Blurred professional boundaries and professional humility positively impacted interprofessional dynamics and student experience in the CLE.

**Discussion:**

This ethnographic exploration of the CLE revealed the emotions‐related processes nested within the interprofessional space of health care practice. In their work towards emotional housekeeping of the CLE, the nurses offer emotional gifts to medical staff and students populating the ward. Workload issues and inadequate staffing influence both cognition and behaviour adversely, impacting interprofessional dynamics. Our findings support fostering professional humility as a pedagogical tool so that we collectively move away from current structures that keep us siloed.

## Abstract 2

Nurses gift emotional support in clinical learning spaces, shaping interprofessional dynamics and student wellbeing–yet stress and silos strain this work. Humility may help bridge the gaps. #MedEd

## 1 INTRODUCTION

Learning medicine is a process of social enculturation that has been widely discussed and explored in contemporary literature. The social enculturation in clinical work‐based setting has a distinct and unique character involving diverse health care professionals and teams. Furthermore, there is a recognition that health professionals' socialisation in the workplace involves an emotional component, including management of feelings as per professional expectations and demands.^1^ In this paper, we aim to investigate how emotions‐related processes among health care professionals in the clinical learning environment (CLE) impact medical students' experiences and socialisation. Illuminating the unexamined emotion schemas in the workplace will expand knowledge on the reproduction of professional behaviours and provide direction to us as a community to develop strategies that enhance interprofessional social exchanges.^2^, ^3^ Additionally, understanding the emotional demands associated with health care practice and education should inform provision of adequate support and supervision for staff and students.

Health professions education (HPE) researchers have formulated emotions as socio‐cultural mediators in addition to internal and universally experienced biological responses and processes.^4^ Emotions are relevant in adapting to both the physical and social realms through modulating our focus and reasoning process.^5^ Recent work revealed medical students' emotionally intense experiences in relation to moral dilemmas encountered in clinical practice and the potential transformative learning.^6^ The resultant implications for student support and faculty development are also highlighted. There is interesting although limited work in relation to emotional responses involved in simulation‐based education and their implications for learning and faculty training.^7^, ^8^ Van Duin et al.^9^ reported that there were emotional responses experienced by junior doctors during interprofessional interactions; they further elaborated on the need for safe spaces to reflect on the experience and modulate their emotions. The findings link to the role of diverse health care professionals, such as nurse educators in the professional development of medical students and junior doctors.^10^

Existing literature mentions the ‘sentimental order’ present in every clinical setting, and the predominant contribution of nurses in maintaining it.^11^, ^12^ The sentimental order implies the intangible pattern of mood that can be disrupted by certain events, and the nurses' work in restoring it in the ward. This chimes with Dowling and Barrett^13^ description of nurses as ‘midwives, delivering doctors safely into their new careers’, implying their critical role in the professional socialisation of newly qualified doctors (p.57). Further, empirical work from multiple clinical sites in the UK describes nurses' contribution to the explicit learning of skills through ‘ad hoc’ encounters in the CLE, capturing doctors' errors with implications for patient safety, and fostering an understanding of professional roles and boundaries.^14^ More recently, multiple dimensions of dyadic interactions of nurses with trainee doctors are suggested in relation to patient safety: nurses as teacher, guardian of patient well‐being, provider of emotional support, provider of general support, expert advisor, navigator and team player.^15^ Thus, it is reasonable to state that nurses' caring role extends beyond patients to include medical staff and students. It is noteworthy though that caring can be considered a conceptually ill‐defined and marginalised activity, frequently undervalued and holding an ambivalent status.^16^, ^17^, ^18^

While there is work discussing emotional aspects of the caring role in HPE, particularly in the nursing literature, it primarily focuses on nurse–patient interactions.^19^ A few selected studies have extended the conversation beyond clinical practice to include caring involved in, for example, interactions with student nurses^20^ or surgeons in the operating theatre.^12^ Although nurses' role in professional socialisation of junior doctors is established, there is limited understanding of the emotionally charged processes involved in the interprofessional educational space of the CLE. There are discussions in higher education broadly regarding compassionate pedagogy as an educational approach that addresses or mitigates distress in the learning environment, emphasising student well‐being and flourishing as key players in the learning process.^21^ This could not be more important in medical education context given that students, and all grades of doctors are known to be at higher risk of anxiety, depression, substance misuse, and suicide than the general public.^22^ Examination of emotion‐related processes among health care professionals was not our primary research question, but rather emergent from a larger study. The original purpose of the research was to explore how different groups of students learn in the CLE, but it became apparent during ethnographic fieldwork that nurses were emotionally available to not just patients but also medical students and doctors, playing a crucial role in diffusing the emotional strain and sustaining the CLE. Hence, the research team undertook a post hoc analysis to illuminate this largely invisible dimension of the nursing role to better understand the emotion work operational in the CLE. Through this exploration, we aim to project emotions as a significant component of learning and socialisation in the CLE, which directly influences the student experience and interprofessional working.

### 1.1 Conceptual framework

The sociology of emotion and the concept of emotion management is based on the premise that social factors influence an individual's emotional expression. The biological, psychological and social nature of emotion was first framed in relation to airline stewardesses, who were expected to smile and ‘be nice’ to passengers (despite not feeling very pleasant on the inside) as a professional obligation.^23^ This ‘commodification of the smile’ signifies emotional demands of public sector work, where employees are expected to work on their emotions, when they spontaneously do not feel them in order to comply with their professional role in the workplace.^18^ Hochschild labelled emotion work for commercial purposes as ‘emotional labour’, which requires ‘coordination of mind and feeling’ (,^23^ p. 7). She further reflected that all relationships, personal or professional, involve emotion work, with individuals or employees ‘doing emotional labour, gladly or reluctantly, brilliantly or poorly’ (,^23^ p. ix). The word `labour’ used in conjunction with `emotion’ emphasises that the caring aspects of profession can be hard and productive work, similar to physical and technical labour; hence, it should be equally valued. Researchers have built on Hochschild's concepts further, labelling the additional gestures of caring in workplace as ‘emotional gifts’; these are not part of the job description but are practised nevertheless.^24^

According to Hochschild, emotion work or labour is subject to social values and influence, and feelings are best understood within the social relational context within which they arise. She elaborates on ‘feeling rules’ as the norms and values concerning feelings that help differentiate between a disapproved feeling and an approved or idealised one.^18^ Feeling rules thereby guide an individual to suppress a socially inappropriate emotion and/or express a socially acceptable one instead. Emotion management is thus a cognitive process interacting with cultural factors and social rules. However, there is scope for emotion to override cognition, creating a possibility of irrational action (in terms of social norms) and volatile expression of repressed emotions.^25^ Furthermore, it is emphasised that emotion management is not a unilateral process but is influenced by the relational aspects of social interactions. For example, the passengers in Hochschild's scenario are not passive participants and would be engendering a variable plethora of feelings among the flight attendants in various social encounters. This interactional and relational attribute of emotional labour and emotional management makes it important to carefully examine inherently social phenomena, such as health care practice and learning.

## 2 AIMS

Positioned in a social constructionist paradigm, we aim to understand the sociocultural determinants of emotions operating in the CLE. Our focus for this exploration is specifically the interprofessional interactions involving nurses that the workplace learning environment entails for medical students and junior doctors. In doing so, we address the research question: How do emotions operate in the informal interprofessional encounters of the CLE, and what are their implications for medical students' experiences in the health care workplace?

## 3 METHODS

Qualitative design is suitable to explore the CLE, which is a socio‐cultural setting with multiple context‐specific realities. Furthermore, an ethnographic approach is considered useful since significant information can be elucidated from mundane events that social actors engage in and ‘thick descriptions have the potential to leak evidence of emotion’ (,^26^ p. 3). Ethnographic enquiries capture the tacit and ‘taken for granted’ dimensions which can yield a rich diction on the emotional dynamics in interprofessional interactions among health care professionals.^27^

### 3.1 Study context

The study was conducted in a Scottish urban hospital, specifically in two teaching wards that hosted clinical placements for fourth‐ and fifth‐year medical students, in the 5‐year long undergraduate medical (MBChB) programme. The criteria specified by Spradley^28^ aided in selection of the fieldsites, where the ethnographer (SG) could study the health professions' world with fresh eyes. The first author (SG) is a staff member in the medical school but has no clinical role in the selected wards, thus enabling a dual insider–outsider perspective, desirable for immersive fieldwork.^29^ Ethical approval for the study was granted by the University of Dundee (UoD) research ethics committee. Additionally, key gatekeepers such as clinical supervisors and nurses in‐charge at the selected wards, along with the curriculum leads, granted access approval to the field. SG located health care staff and medical students during initial fieldwork, shared details of the research and provided clarifications as required. Consenting individuals were shadowed during non‐participant observations in the wards. It is noteworthy that patients were not included in the study and did not form part of any observations.

### 3.2 Sampling and data collection

A combination of purposive and convenience sampling was employed to recruit medical students, foundation doctors (junior doctors in the UK), postgraduate trainees, consultant supervisors and other health care professionals such as nurses and pharmacists.^30^ Written informed consent was obtained from participants for observations and interview, including recording and dissemination in deidentified format. Observations (120 hours) were conducted in the two selected wards over a period of 10 months, focussing on the mundane features of `everyday life’ in the CLE that were relevant for student learning and overall experience. Once embedded in the social life of the CLE, SG got exposed to the negotiated nature of life and work in interprofessional health care teams. Handwritten fieldnotes were taken contemporaneously during observations, which were expanded and typed up to create a digital record of the reconstructed reality of the CLE.

In situ observations helped in recruiting key informants, who were health care staff and students with whom SG associated closely in the field, and developed trust and rapport.^29^ Ethnographic interviews were conducted with 36 key informants, and these helped inform observations further. The interview schedule varied for each participant and was derived predominantly from the observations in situ but also partly from the research objective and literature review. It included prompts to understand the range of student experiences in the CLE, the hierarchical issues and the interprofessional interactions with diverse staff. The primary objective was to glean a diverse and holistic perspective of participants' life in the CLE, adopting a flexible and reflexive approach. Interviews were audio‐recorded and transcribed manually. Data from fieldnotes and interview transcripts were stored and managed as per the University of Dundee's research protocol (Data protection | University of Dundee, UK) and the NVivo 12 Plus software.

### 3.3 Data analysis

**FIGURE 1:**
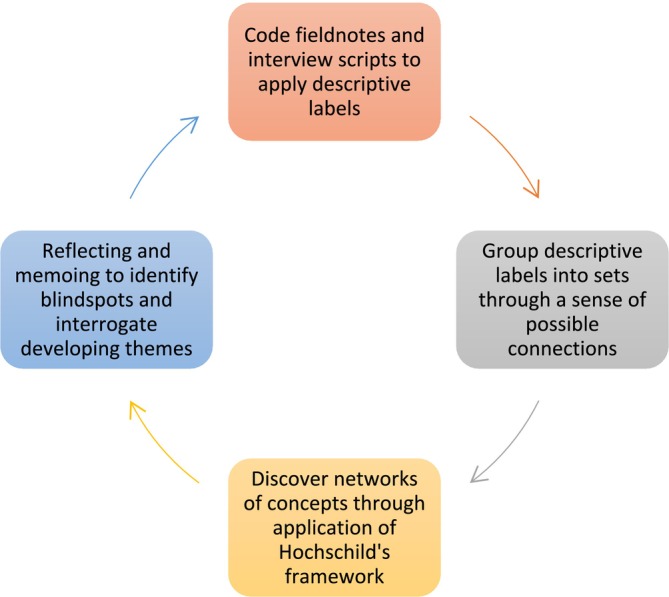
The circular process of ethnographic data analysis repeated iteratively (adapted from Roper and Shapira^31^). [Color figure can be viewed at wileyonlinelibrary.com]

Figure 1 highlights the circular process of data analysis which was followed based on the guidance by Roper and Shapira,^31^ around handling bulky ethnographic records in a systematic, although non‐linear fashion to convert emic information to etic understanding. This is a rather simplistic illustration of our efforts to decode the community's culturally coded messages using the sensitising concepts of emotional labour, emotional gifts and feeling rules, together with the relational and interactional dimensions of interprofessional interactions in the CLE.^23^ Data from field observations and interview transcripts were read and re‐read to exhaust potentialities of the vast data gathered and to chunk data into meaningful pieces to which descriptive labels could be assigned. Further sorting and sifting helped to identify patterns in the labels allowing incorporation into a model, while balancing both emic and etic perspectives. Application of Hochschild's theory while viewing the mundane and taken for granted practices in the CLE aided in making overly familiar data look alien and an additional comprehension of participants' life. The authors engaged deeply with the participants' stories to trace similarities and differences, paying attention to contradictions in the data in relation to the research question and reduce the complexity without violating it.^27^ All members of the research team compared their interpretations and interrogated contextual factors that explained the themes and their relationships. Frequent deliberations were held to expand upon contingent mechanisms, incorporate these dimensions into the end‐stage analysis and reach consensus on the final themes.

### 3.4 Reflexivity

We maintained an awareness of our impact on the research process and adopted a reflexive approach throughout data collection, analysis and writing to enhance the credibility. Our research team comprised of a psychologist, a social scientist and educators with past and current experience of health care practice. All members identify as women, and one researcher would be categorised as an ethnic minority in the UK. We believe that complementary expertise facilitated through this diversity within the research team was especially valuable in challenging assumptions and creating a unique intellectual blend. Our foremost concern was to depict the reality of the fieldsites honestly through combining the emic (natives or participants) and the etic (outsiders or researchers) perspective in a meaningful manner, in keeping with ethnographic tradition.^32^ In addition to team meetings, reflective journals and analytic memos ensured validity and trustworthiness of our findings. Member‐checking was possible with participants who were the health care staff in the fieldsites; however, medical students and junior doctors were difficult to follow‐up after they had left the field‐sites owing to short and rotational placements in the CLE.

### 3.5 Findings

**FIGURE 2:**
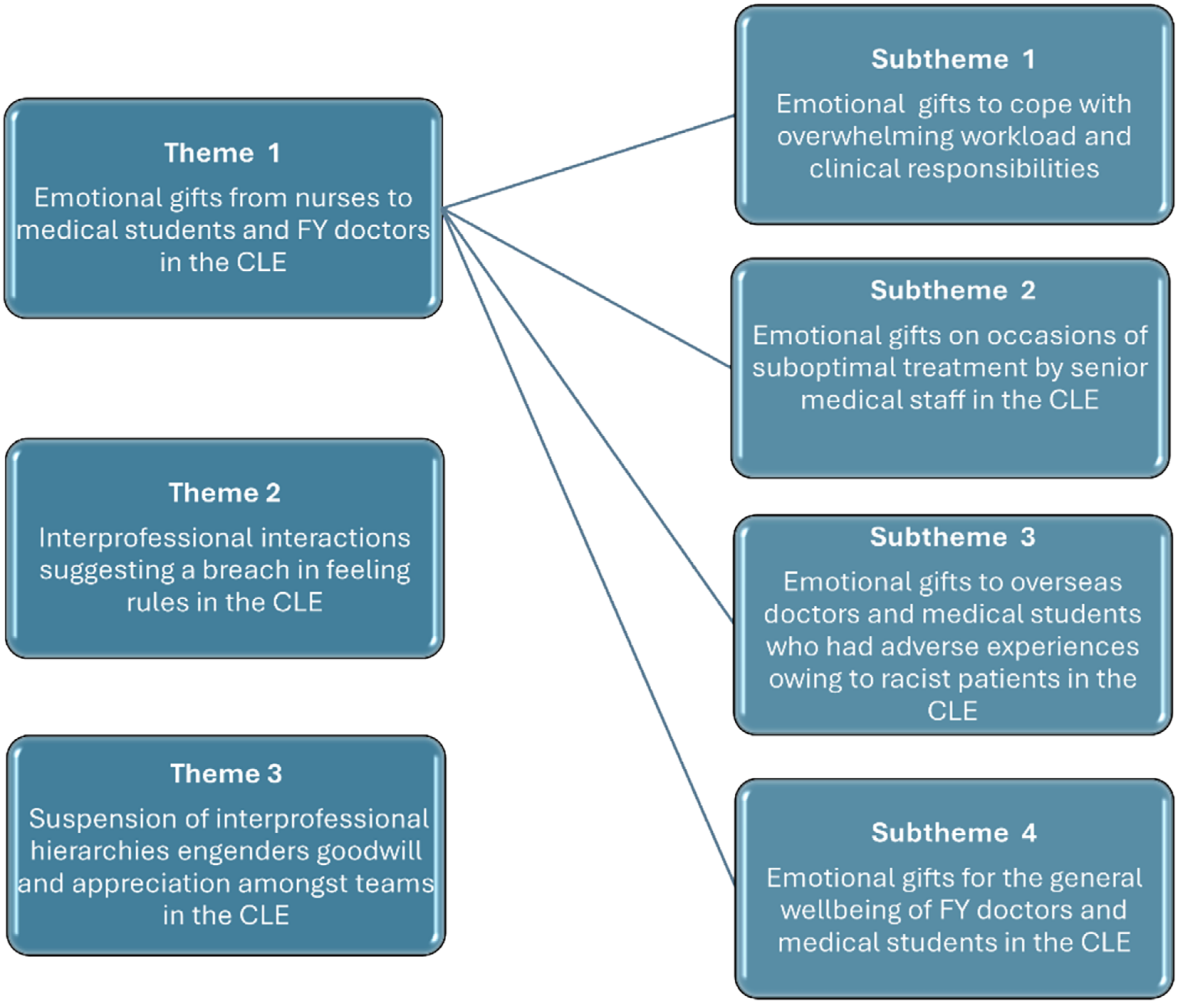
Key themes and subthemes derived from data using Hochschild's framework (CLE, clinical learning environment; FY, foundation year [junior doctors in the UK]).

Key themes related to the emotional dynamics in interprofessional interactions in the chosen hospital wards are presented below (Figure 2). These are derived based on the Hochschild's conceptual framework on emotional labour. The themes are supported by fieldnote extracts and sections of interview transcripts to convince the reader of the reality in the CLE as apparent to the ethnographer (SG). It is noteworthy that certain sections are replaced as to protect the identity of the fieldsites and the research participants.

Consenting participants are assigned identity codes, such as CS for Consultant Supervisor, PT for Postgraduate Trainee, FY for Foundation Doctor, MS for Medical Student, WN for Ward Nurse and HP for Hospital Pharmacist. Please see Table 1 for the number and list of study participants who were health care staff and students populating the fieldsites.

**TABLE 1:** Number and sources of interview data.

Participant group	Number
Medical student (MS)	13
Foundation doctor (FY)	8
Postgraduate trainee (PT)	6
Consultant supervisor (CS)	5
Ward nurse (WN)	3
Hospital Pharmacist (HP)	1
**Total**	**36**

**FIGURE 3:**
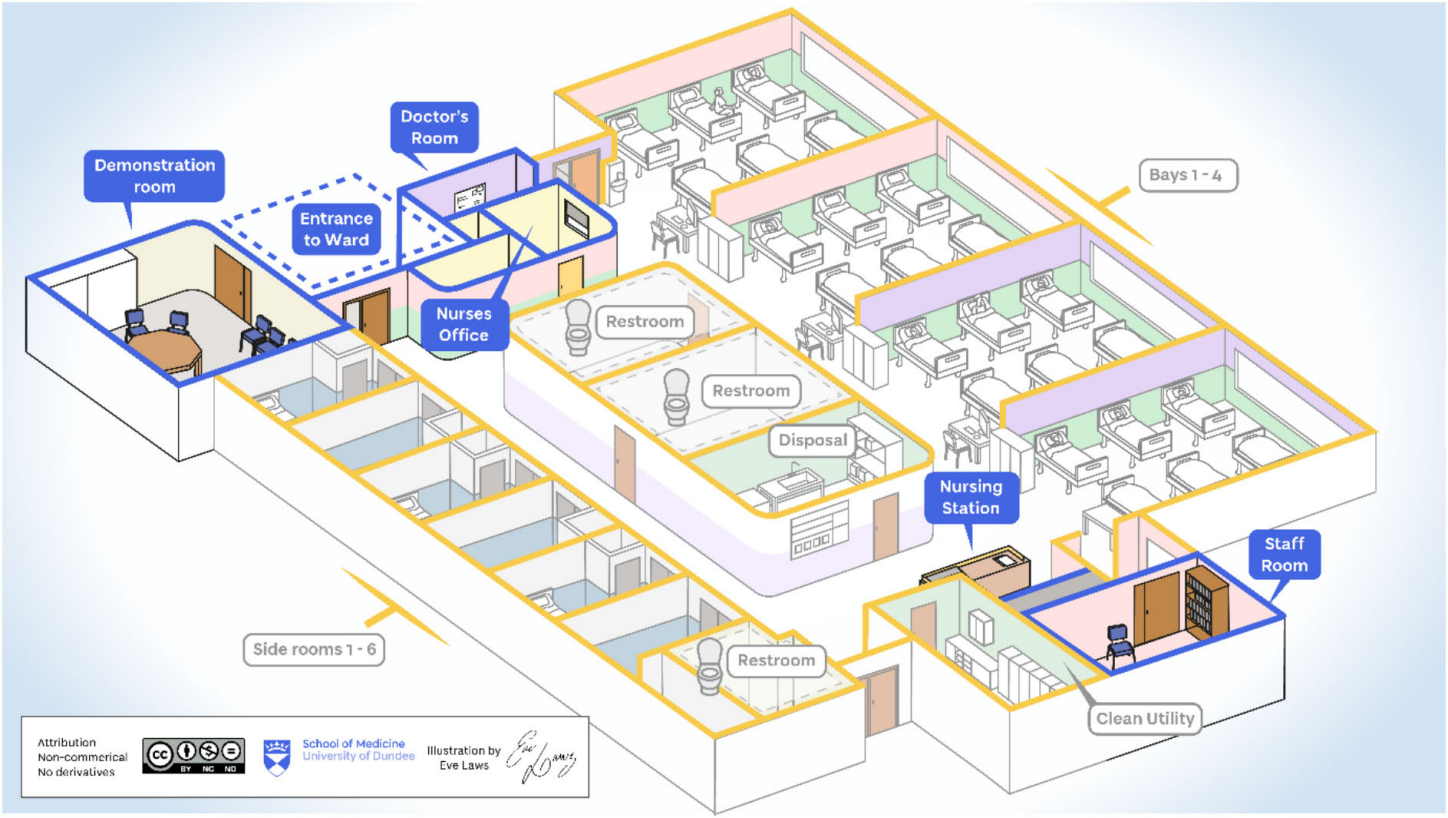
Research fieldsite 1–30‐bedded medical ward.^33^ [Color figure can be viewed at wileyonlinelibrary.com]

**FIGURE 4:**
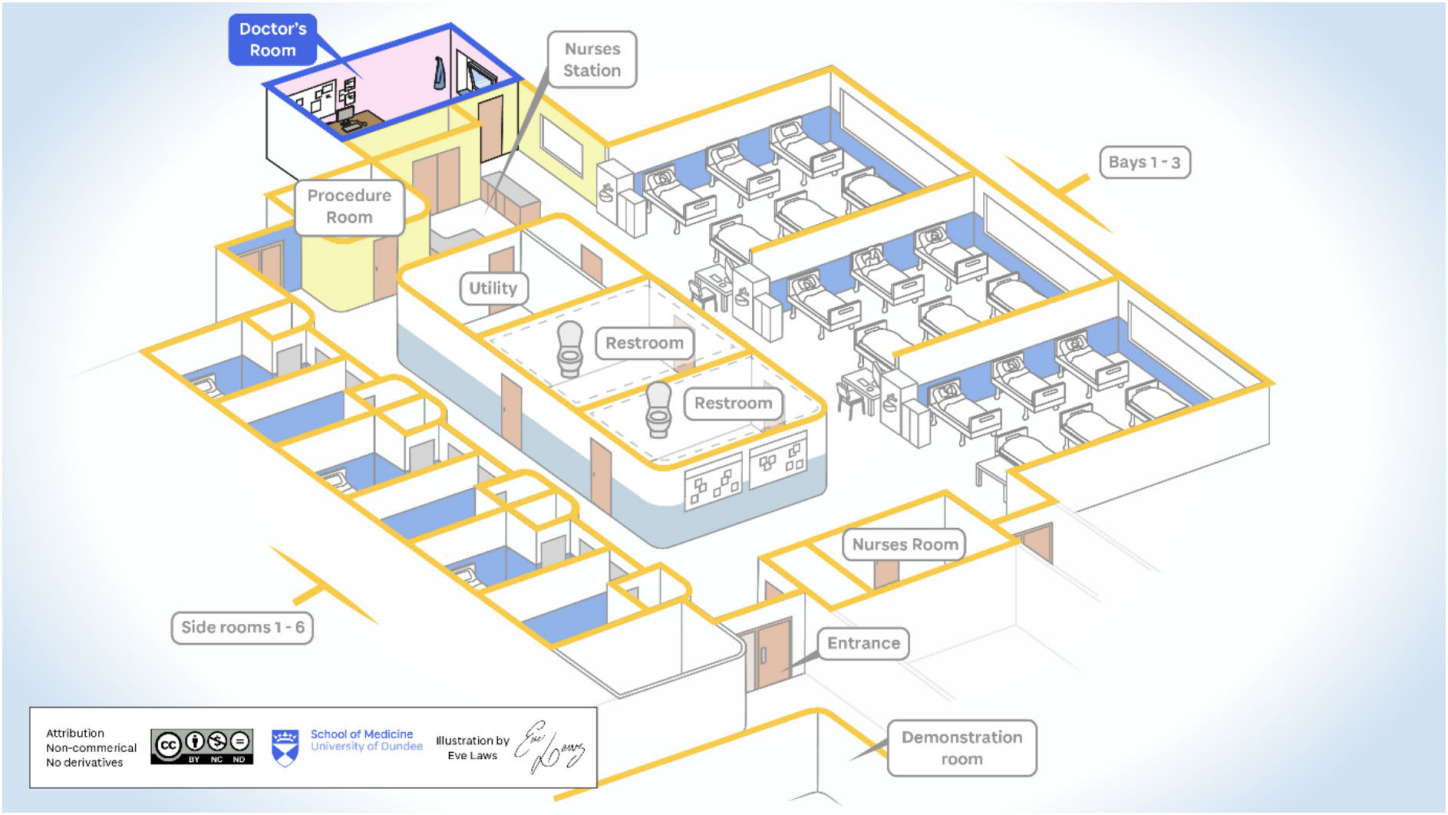
Research fieldsite 2–24‐bedded surgical ward.^33^ [Color figure can be viewed at wileyonlinelibrary.com]

Figures 3 and 4 are illustrations of the two selected wards where fieldwork was conducted, which should aid the reader in understanding contextual descriptions and the themes discussed below. These have been created by the medical school artist, as the ethnographer did not take any pictures of the clinical sites for ethical reasons.

### 3.6 Theme 1: Emotional gifts from nurses to medical students and FY doctors in the CLE

The data indicate that staff from the nursing team in both the fieldsites frequently provided comfort and support to medical students and junior doctors. These gestures of caring may be characterised as gifts, as they are not core patient care related tasks but rather indications of good practice to support learners and junior doctors. The following subthemes reveal the underlying reasons which led to emotional burden and stress for students and FY doctors in the wards. The nurses would strive to maintain the sentimental order in the CLE through generously gifting emotional support to those needing it, as evident in the stress provoking scenarios described below.

#### 3.6.1 Subtheme 1. Emotional gifts to cope with overwhelming workload and clinical responsibilities

Study participants reported that FY doctors felt less prepared for clinical work expected of them in the workplace, and it would be a frequent source of stress leading to emotional breakdowns on occasions. The following medical student describes how she had witnessed junior doctors crying and seemingly burnt out, owing to inability to cope with duties and demands of busy clinical workplaces.

MS8: *“..the emotional toll it takes, FYs being burnout out and crying because of the stress of the job like made a mistake, or a patient was mean, or on reflection they could have handled a situation differently [.] “”ohh. I've had the worst night ever, I cried twice on the night duty!”” Another time it was an FY on oncology who was overwhelmed, [.] The nurse on the ward supported him and forced him to take a break and go out.”*

The stress related to clinical duties and how it unsettled FY doctors was also alluded to by the nurses on the chosen wards, who appeared sympathetic towards the junior doctors.

WN 2: “*FYs are not ready when they come to us, they are overwhelmed by the workload. I had an FY1 crying the other day during my evening shift. She was having to cover several wards on her own, nearly 60 patients. [.] We try and help them out as best as we can, in managing clinical tasks as well as support emotionally.”*

#### 3.6.2 Subtheme 2: Emotional gifts on occasions of suboptimal treatment by senior medical staff in the CLE

Study participants narrated instances when seniors such as consultants demonstrated lack of respect for junior doctors, which led to emotional stress for them. According to participants, they could be at the receiving end of the senior's wrath for seemingly trivial lapses and the nurses on the ward would provide support and comfort on such occasions.

FY4: “*I've experienced consultants or senior doctors shouting in the middle of a ward in front of patients. I feel perilous in those situations, can't think straight. By the time you sort of process that, it's over and you're sort of why would that be happening? It was something about a discharge letter, something relatively easy to fix I would say [.] No danger to patient or anyone's life! The consultant came in and swore quite loudly in the ward. [.] I was sounded off even though I wasn't at fault per say, but I was the sort of the generic FY to shout at [.] We don't need to be shouted at for the message to be put across. It's scary! Disproportionate reaction for whatever reason. It wasn't appropriate how it looked to patients. The nurses on the wards are a huge support in such instances, don’t know what we would do without them*”.

The student participants also reported similar experiences when they felt targeted and disrespected by the consultants for their lack of knowledge related to the speciality. These situations were even more hurtful if they took place in the patients' presence, and again, the ward nurses ensured the targeted student's well‐being in such scenarios.

MS7: *“I've had a couple of examples of, maybe not knowing something. And the consultant saying very rudely “” how do you not know this?”” in front of the patient and the patient later on in the day came up to me and said “” I'm sorry that they spoke to you like that””. That has happened on more than one occasion when I've left the wards very upset. There was the staff nurse around once and she was frequently checking up on me”*.

MS11: *“in the OT (operation theatre), there was a patient with uncal herniation and I got excited because that's a textbook thing, right? You don't really see it everyday [.] The consultant and registrar were discussing the case, and I joined them to hear what they were talking. The surgeon shouted rudely at me “You think we are doing this for fun, it is patient safety we're talking here””. And he asked me to get lost. I don't think I was disruptive or intrusive in any way, just curious and interested in learning about the case. And at the point in time, he wasn't even doing anything with the patient. He was just briefing his registrar. I went away in a corner, quite shocked and almost paralysed. The theatre nurse came to me and said‐ “” I want to apologise for his behaviour. He doesn't usually behave like that [.]””. She was comforting, I felt better but as a student, we do not necessarily feel fully safe in our position”*.

The nurse participant in the study expressed confidence in challenging senior medical staff regarding their suboptimal behaviour, as expressed below. It was evident in the data that nurses play the sentinel role in the clinical sites, and they considered it their responsibility to maintain the sentimental order; thereby, they were able to exercise authority over the conduct of those populating these sites.

WN1: “*Sometimes the consultants will have a dig at the FYs if the jobs are not done properly. Some consultants can throw a fit, I will tell them off for it! [.] I wouldn’t be tolerating bad behaviour on my ward. My friend is in the theatre, and she is very sharp if the surgeon is throwing a tantrum”*.

#### 3.6.3 Subtheme 3: Emotional gifts to overseas doctors and medical students who had adverse experiences owing to racist patients in the CLE

Both the selected wards had International Medical Graduates (IMG) working as FYs, trainees and consultant doctors, as well as overseas medical students. During fieldwork, the ethnographer (SG) observed an instance when the staff nurse purposefully visited the doctor's room to warn the medical staff and students regarding a racist patient.

Fieldsite 2: This morning, *FY4 and FY5 (IMG) are the junior doctors on duty, and they are discussing a patient's management with PT5 (IMG). MS6 and MS13 (ethnic minority student) are studying a patient's file too, I recall they have a case presentation in the afternoon. WN3 pops her head in the room and says in a hushed tone “just to warn you, the patient in side‐room 2 is very racist, I don't want you going near him. The white friends will manage this one”*.

The participants reported how racist slurs from patients were a common occurrence in the CLE, and the ethnic minority staff and students would make their seniors aware of this. Apart from urging patients to be respectful towards those caring for them, there was little that could be done regarding the issue as health care staff continued to care for sick patients regardless of their negative behaviour. There were, however, frequent instances of white allyship reported among the medical and nursing staff who felt protective towards their minority colleagues.

PT5 (IMG): “*..the patient refused to be touched by me, kept insisting on a white doctor [.] racism on the wards is a sad reality![.] It happens at least twice a week on the average. [.] I've gotten good support from a few of the local nurses. A senior ANP (advance nurse practitioner) has given me a third person perspective on several matters and how the system works and occasionally personal life as well. I would say that I am doing well all thanks to her*”.

MS2 (ethnic minority medical student): “*I went with the canulation tray to this lady in bay 4. She kept muttering rude and racist comments, wincing and giving me looks of disgust throughout. It was distressing and demeaning. I didn’t realise but a nurse had overheard, she came up to me and asked if I was okay, and if I would like to escalate it, which I found pretty supportive”*.

#### 3.6.4 Subtheme 4: Emotional gifts for the general well‐being of FY doctors and medical students in the CLE

Over 120 hours of fieldwork in the selected wards afforded the ethnographer several opportunities to observe the caring role of the nurses towards the medical students and junior doctors. SG noted numerous instances when WN2 in fieldsite 1 and WN3 in fieldsite 2 were approached whenever any medical staff or student needed to clarify an issue related to patient care, the general functioning of the ward or any wider subject such as procurement, pharmacy deliveries or an investigation. Inevitably, these nurses were able to solve the problem or signpost to the solution. They appeared reliable and responsible, and their efficient and confident demeanour reminded the ethnographer of a ‘maternal presence’ in the ward. The quote below from a senior postgraduate trainee who had been working with WN2 for several years further reinforces this.

PT3: “*WN2 keeps everyone right, but I must admit I am a bit scared of her as well, I will get a telling off if I am careless (laughs)*.”

In a similar vein, the senior nurses described how they made sure that all clinical duties and patient‐related tasks were executed in an organised and timely manner in their wards.

*WN3: “Both FY4 and the 2 girls are very hardworking. Previously, we had FY7, who was great too. FY6 was a bit cheeky but we sorted him out. He knew his stuff, don’t get me wrong there, but he would try to sweet talk himself out, like leave jobs for the FYs coming in the next shift. But I did not let him get away with that and made sure that he finished before leaving and did not create problems for the next shift”*.

A recurrent pattern of nurses' caring behaviour extended beyond patients, to ensure that junior doctors went for their lunch breaks. The reader must take note of the following extract from the fieldnotes not as an isolated incident, but a regular occurrence observed during fieldwork.

**Fieldsite 1:** WN1 is discussing a patient's medication with FY3, and as she leaves, she reminds FY3 to go for her lunch. She says “Go get proper lunch, I see you snacking on some piece of junk always. That's not good. Have a proper break, make sure to carry your bleep, we will manage while you are away. And if there is anything I will bleep.”

The nurses on the selected wards also supported the students, when they struggled to cope with the emotional consequences of clinical work, such as students breaking down owing to a deteriorating patient or a medical emergency in the ward.

WN3: “*It is easy for some, and some take it very hard. Only yesterday, we had (medical student) who was nice but is a softie (sensitive and soft‐hearted person). We had an arrest in the ward whilst he was here [.] We were all busy on autopilot mode. After getting things sorted and transferring the patient, when I was catching my breath, I see him sitting in a corner and crying. It is difficult, they are young. We are used to it, we see lots of arrest, deaths, everything. I told him to cry it off, no need to bottle things up. I also told him to visit the chaplaincy, they are good too, they provide support. We all need support from time to time*”.

### 3.7 Theme 2: Interprofessional interactions suggesting a breach in feeling rules in the CLE

According to the study participants, the vast majority of the interprofessional interactions were pleasant and positive. However, there were a few instances reported where medical students and FY doctors felt targeted by the nurses in the wards. The ‘feeling rules’ according to the social norms in the CLE require interprofessional respect and supportive interactions between professional teams. These feeling rules are occasionally bypassed as per the participants' experiences below.

MS9: “*Some nurses are hypocritical, for example, last week after taking bloods for a patient and cleaning up, I was chatting with them. I might have accidentally knocked the alcohol wipe wrapper off my tray, which I didn’t notice. The wrapper was on their bed, and I was about to walk away. When the ward nurse pulled my arm, and said loudly ‐ “” What do you think you're doing? It is unprofessional to litter patient’s bed, and you shouldn't be taking up their personal space with medical equipment. Leave my ward now!”” And I was left thinking, I'm sorry, I agree. I shouldn't have dropped it. No argument about it. But I didn't mean to, it fell off my tray. If I'd left the needle on a bed, I would understand this reaction.”*

FY6: “*It's almost as if some nurses are waiting for someone to make a mistake to jump at it. That's something you can sense because you can feel that all eyes are on you, and you can judge it by the proportion of the reaction to something very minor”*.

This breach of social norms regarding the interprofessional interactions appear to be linked to stresses in the CLE such as workload and under staffing. Additionally, there are certain times in the day when the teams are stretched, and the presence of medical students can be deemed obstructive to carrying out clinical duties as highlighted below.

WN1: “*I can be snappy, if a student starts asking questions at 8am or 9am, there are so many jobs needing done [.] Nursing is severely understaffed. People are burnt out and calling in sick every other day. That creates pressure. The newcomers are meant to have a senior mentor them, and a proper induction, but that is not happening. They are just thrown in from day one, to sink or swim. Same with the doctors too. And that's what leads to problems, people being burnt‐out, doctors phoning in sick. One person is doing the workload of three people. You can request the rota coordinator that you need help. But if that help doesn't come immediately, you're still faced with all the patients”*.

The ethnographer did not observe any overt targeting of medical students or FY doctors during field observations, but there were instances of subtle undermining behaviours by nurses, which appeared to be gender‐related; these are reported elsewhere.^34^

### 3.8 Theme 3: Suspension of interprofessional hierarchies engenders goodwill and appreciation among teams in the CLE

Data from fieldnotes and interview scripts suggested that there was a variation in interprofessional dynamics among different health care staff and students, implying that some doctors and medical students got along better with the nurses than others. Nurses appeared to like doctors that displayed less hierarchical behaviours and more actions suggestive of interprofessional humility. The following fieldnote extract illustrates that a consultant physician, who came across as one of the most popular senior doctors on the ward would not shy away from doing any patient care related tasks.

**Fieldsite 1:** *At the nursing station today, while I was chatting to WN1, a porter arrives to fetch a patient to the radiology department for an investigation. CS2, (consultant of the week) is with his medical team doing the ward rounds remarks – “oh great, the porter is here, the patient can go down for the scan now” and he spontaneously went off to get her a gown that she could change into for the procedure. It is interesting because most other consultants would not have done this kind of thing. Most folks would leave it for the nurses to sort these jobs. It is perhaps these little gestures reflecting humility that make him so likeable to everyone*.

Field observations in the two selected wards indicated that the CLE was a hierarchical environment in terms of interprofessional dynamics, although there was variation in the degree displayed by different health care professionals. The ethnographer (SG) perceived more collegiality and respect when there was crossing of interprofessional boundaries. This was reported by medical students as well in the interview transcripts below.

MS3: “*is my role model, she is just so efficient and very popular with the nurses. Last week a patient deteriorated, and the nurse was kind of beating herself up because she hadn't given her medication exactly on the time. It was a very busy day, and everyone was being rushed off. The doctors didn't think the patient's deterioration was because of that medication was giving the nurse a hug and consoling her*”.

MS6: *“The nurses are very fond of PT1. He addresses the nurses by their names and always thanks them for whatever they might have done for the patients. Just the way he speaks to them [.] he did the checks himself, which technically would be a nursing job. Things like that make him very popular, quite understandably. And the way he kneels at the patients' bedside to talk to them at eye level, it has an altogether different impact. Not all senior doctors are like this. You can tell when the doctors are like almost dismissive of the nurses and then nurses don't like the doctors either.”*

## 4 DISCUSSION

This paper aimed to tease out the emotional dynamics operating in the informal interprofessional educational space of the CLE. Our findings establish that nurses provide emotional support to medical students and junior doctors in a variety of situations, impacting their professional socialisation and well‐being. Employing Hochschild's framework helped elucidate the relational elements involved and explain contextual drivers to social tensions in the CLE.

Over 30 years ago, Dowling and Barrett^13^ advocated for nurses' role with junior doctors to be formally acknowledged, and more recent studies have further reinforced the importance of their contribution.^14^, ^15^ Our findings confirm that the somewhat unpaid mentoring and pastoral care delivered by the nurses have not diminished, and rather the nurses in the CLE contribute to the induction and holistic functioning of students and junior doctors. Our theory‐informed approach helped to untangle the subconscious processes intrinsic to the interprofessional interactions in the CLE. There are suggestions in our data that junior doctors and medical students could be in emotionally vulnerable positions in the CLE, on account of workload and suboptimal treatment by seniors or patients. The nurses' caring and emotional housekeeping work towards ensuring their well‐being was evident in the data. As established in existing literature though, little ‘gestures of caring’ or emotional gifts can go unnoticed in the service‐oriented ward setting, and they continue to be undervalued and kept on the margins of the formal and timetabled teaching.^20^, ^24^

Previous work described nurses' emotional work in an operation theatre as the ‘hostess role’ keeping everything in order and everyone happy.^12^ This appears analogous to the nurses' maternal role towards students and staff in the CLE in our study. Our findings also resonate with reports of health care staff and students' mistreatment by patients.^35^ We additionally found that the nurses on the wards provided comfort and emotional assistance to the victims in these instances. Participants in our study shared experiences of humiliating treatment by senior medical staff, similar to those highlighted by Leedham‐Green et al.^36^ Nurses' emotional support and caring was appreciated by the junior doctors and students as a stabilising factor in such situations. In highlighting the caring role towards medical students and junior doctors in the CLE, we advocate for an explicit recognition of this dimension of nurses' professional role. Literature frequently notes the entrenched hierarchies and implicit status differentials with regard to nurse practitioners and doctors that pose obstacles to positive interprofessional culture.^9^, ^37^ Awareness within the HPE community of the positive and nurturing impact of the nurses on other professions in the CLE (even when they have no specific or formalised educational role) may generate goodwill and collaborative working among all professional groups. Moreover, the nurses' caregiving role helps in realising the full potential of work‐based learning, given that student learning is negatively impacted when their well‐being is threatened, as per compassionate pedagogies principles.^38^, ^39^

Participants in our study described how time pressures and staff shortages appeared to impact interprofessional dynamics negatively, driving some nurses towards impatient and irritable behaviour. Overall, reports of positive interprofessional behaviour outnumbered the negative instances, with participants expressing mutual respect and appreciation of each other's contribution towards patient care. Our data regarding workplace stresses resonate with the recent General Medical Council (GMC) report stating that 43% of doctors reported finding it difficult to deliver high‐quality patient owing to workload and inadequate staffing (Workplace report ‐ GMC). Discussions in the literature of acute and problematic interprofessional situations involving medical students are not new.^36^ Addressing these tensions requires consideration of emotions generated, given that emotions are contagious among individual team members and have the potential to influence both cognition and behaviour.^40^ It is reassuring to note in our data that medical students are able to gauge contextual factors and learn constructively from both positive and negative experiences.

An interesting facet of our findings relates to the display of interprofessional humility by senior medical staff, and this was observed by the ethnographer in the field as well as confirmed by medical students in the interviews. The goodwill engendered through suspension of professional classes and confines in work chimes with the recent conversations around professional humility.^41^ The embedded profession‐specific status hierarchy within health care teams has been criticised as a barrier towards interprofessional collaborative practice.^42^, ^43^ Generally, secondary care settings are known for a more hierarchical structure with rigid status differentials as compared to primary care settings.^44^ The blurred professional boundaries revealed in the present study and its positive impact on interprofessional dynamics and emotional order of the CLE suggest that there is value in fostering professional humility among health professions learners in their preparation for clinical practice. So far humility as a construct has primarily been discussed in HPE realms through editorials and opinion pieces, as fostering egalitarian interactions among teams alongside better care coordination.^41^ There is scant empirical evidence around the longer‐term impact of the few humility‐building programmes that exist, such as Interprofessional Training in Empathy, Humility, Affect, and Mindfulness (I‐TEAM) and Cornell University's cultural humility training.^45^ We believe the saturated nature of health professions curricula may be a barrier in active uptake of these courses.

## 5 LIMITATIONS AND FUTURE DIRECTIONS

Our sample perhaps could have been more balanced to have a stronger nurse participants', pharmacists and other health care professionals' and the senior medical staff voice; as such, our themes are reflective heavily of the students' and FY doctors' perspectives based on their everyday interactions in the CLE. It would be worthwhile to explore the subject with significantly larger number of nurse participants, to deeply understand their perspective on the caring and emotional housekeeping that the nursing workforce shoulders in supporting the doctors and students on the wards. Future research can also focus on the nurses' contribution to the hidden curriculum specifically, given that emotional well‐being in the CLE cannot be divorced from learning. We acknowledge that our empirical data shed light on only two (of the many) professional groups in the multidisciplinary health care team. However, nurses and medical staff and students happened to be the majority of the ethnographic hosts in the two fieldsites and were the key informants as opposed to other health care professionals (pharmacists, dietician, physiotherapists) who stood out as occasional and brief visitors to the ethnographer during fieldwork. Future studies can perhaps include wider health care professional groups and settings. An ethnographic study can claim limited generalisability, and our projections should be viewed with caution, given that they stem from two specific wards from a single institution. However, we hope that data triangulation from fieldnotes and interviews, as well as paying close attention to contextual insights by a diverse research team, should mitigate this to an extent.

## 6 CONCLUSION

Our findings highlight the emotions‐related processes and mechanisms nested within HPE and practice, most notably those impacting interprofessional space in the CLE. It is argued that caring is of central value in professional nursing, and nurses include caring for medical students and doctors as an extension of their role. Through an ethnographic exploration of the CLE, we reveal a hitherto tacit emotional dynamic in the interprofessional interactions. In their work towards emotional housekeeping of the CLE, the nurses offer emotional gifts generously to health care staff and students populating the ward. However, workload stresses and understaffing are contextual challenges that can precipitate occasionally negative interprofessional experiences. In highlighting the emotional topography of the CLE, we invite health professions educators to consider fostering professional humility as a pedagogical tool so that we collectively move away from current structures that keep us siloed.

## AUTHOR CONTRIBUTIONS

**Shalini Gupta:** Conceptualization; investigation; funding acquisition; writing—original draft; methodology; writing—review and editing; formal analysis; project administration; data curation. **Stella Howden:** Conceptualization; investigation; funding acquisition; methodology; writing—review and editing; formal analysis; project administration; supervision. **Mandy Moffat:** Validation; writing—review and editing; formal analysis; supervision. **Lindsey Pope:** Validation; writing—review and editing; formal analysis; supervision. **Cate Kennedy:** Conceptualization; validation; formal analysis; supervision.

## CONFLICT OF INTEREST STATEMENT

The authors disclose no conflicts of interest.

## Data Availability

The data that support the findings of this study are available on request from the corresponding author. The data are not publicly available due to privacy or ethical restrictions.
